# Thyroid Hormone Treatment and Breast Cancer Risk in Women: A Systematic Review and Meta-Analysis of Observational Studies

**DOI:** 10.3390/metabo16070465

**Published:** 2026-07-02

**Authors:** Stylianos Kopanos, Jasper David Feldkamp, Johanna Tyssen, Xinjun Li, Kristina Sundquist, Carolina Pape-Köhler, Marcel Binnebösel, Annika Hoyer, Per Wändell, Joachim Feldkamp

**Affiliations:** 1 Academic Department of General Internal Medicine, Endocrinology, Diabetes and Infectiology, Medical School and University Medical Center East Westphalia-Lippe, Bielefeld University, 33604 Bielefeld, Germany; johanna.tyssen@uni-bielefeld.de (J.T.); joachim.feldkamp@klinikumbielefeld.de (J.F.); 2 Division of Hematology, Oncology, and Cancer Immunology, Charité—Universitätsmedizin Berlin, Corporate Member of Freie Universität Berlin, Humboldt-Universität zu Berlin and Berlin Institute of Health, 10117 Berlin, Germany; jasper.feldkamp@charite.de; 3 Center for Primary Health Care Research, Lund University, 202 13 Malmö, Sweden; 4 Department Family Medicine and Clinical Epidemiology, University Clinic Primary Care, Skåne University Hospital, 205 02 Malmö, Sweden; 5 Klinikum Bielefeld—Rosenhöhe, Bariatric Clinic, 33647 Bielefeld, Germany; 6 Academic Department of Surgery, Klinikum Bielefeld, Medical School and University Medical Centre East Westphalia-Lippe, Bielefeld University, 33604 Bielefeld, Germany; 7 Biostatistics and Medical Biometry, Medical School OWL, Bielefeld University, 33501 Bielefeld, Germany; 8 Division of Family Medicine and Primary Care, Department of Neurobiology, Care Sciences and Society, Karolinska Institutet, 141 57 Huddinge, Sweden

**Keywords:** thyroid hormone treatment, levothyroxine, breast cancer, hypothyroidism, systematic review, meta-analysis

## Abstract

Objective: Thyroid hormone treatment is the standard therapy for hypothyroidism, particularly in women. Concerns have been raised that exogenous thyroid hormone use may increase breast cancer risk, but evidence remains inconclusive. This study aimed to systematically review and synthesize observational evidence on the association between thyroid hormone treatment and breast cancer risk in women. Design: We conducted a systematic review and meta-analysis of observational studies. Methods: MEDLINE, EMBASE, and Web of Science were searched from January 1976 to February 2025. Eligible studies assessed breast cancer incidence in adult women receiving thyroid hormone treatment versus non-users. Pooled ORs were calculated. Findings from cohort studies reporting hazard ratios were synthesized qualitatively. Heterogeneity, publication bias, and certainty of evidence were assessed. PROSPERO ID: CRD42022348966. Results: Four case–control studies including 221,254 women receiving thyroid hormone treatment and 4,385,666 controls were included in the prespecified primary OR-based meta-analysis. In the primary random-effects meta-analysis, thyroid hormone treatment showed a possible epidemiological signal with breast cancer risk (OR 1.43, 95% CI: 0.90–2.28; I^2^ = 94.3%), although the confidence interval crossed unity and heterogeneity was substantial. Formal assessment of publication bias was performed but should be interpreted cautiously given the small number of included studies. The certainty of evidence was rated as low due to heterogeneity, serious inconsistency and imprecision. Conclusions: Thyroid hormone treatment was associated with a possible epidemiological signal for breast cancer in observational studies; however, the primary pooled estimate was not statistically significant and should be interpreted as hypothesis-generating because of substantial heterogeneity and residual confounding. Further well-designed prospective studies are required before causal or clinical inferences can be made.

## 1. Introduction

Hypothyroidism is one of the most common endocrine disorders worldwide, disproportionately affecting women and older adults. Its standard treatment involves long-term administration of thyroid hormone replacement therapy, primarily levothyroxine (L-T4)—a synthetic analogue of thyroxine. While the primary therapeutic aim of thyroid hormone replacement is to restore euthyroidism by substituting thyroid hormone, thyroid hormones also play broader roles in systemic physiology, including regulation of cellular proliferation, differentiation, and apoptosis—pathways centrally involved in carcinogenesis [1].

Breast cancer remains the most commonly diagnosed cancer in women globally and is a leading contributor to cancer-related mortality. As such, understanding modifiable or iatrogenic risk factors is critical for prevention strategies. Given the high prevalence of thyroid hormone use, even a modest increase in breast cancer risk associated with levothyroxine exposure could have significant public health implications [2,3]. Mechanistic studies have demonstrated that thyroid hormones may exert oestrogen-like effects on breast tissue through genomic and non-genomic mechanisms. Specifically, thyroid hormone receptors (TRs) may interact with oestrogen receptor signalling pathways, potentially enhancing estrogenic stimulation and promoting tumorigenesis in hormone-sensitive tissues [4].

Despite biological plausibility and accumulating observational research, epidemiological findings on this association remain inconsistent. While some studies suggest a positive association between thyroid hormone therapy and breast cancer risk, others report null or even inverse relationships. These discrepancies likely stem from methodological heterogeneity across studies—including variation in study design, population demographics, duration of follow-up, hormone formulations, and the extent of adjustment for potential confounders [5,6].

Importantly, no comprehensive meta-analytic synthesis has yet addressed this question using a rigorous and transparent methodology. Given the absence of randomized controlled trials (RCTs), a systematic review and meta-analysis of observational data—appropriately stratified by effect measures and assessed for bias and heterogeneity—provides the most reliable available evidence [7,8].

Levothyroxine is among the most frequently prescribed medications in industrialized countries due to the lifelong nature of treatment and increasing rates of diagnosed thyroid dysfunction [9,10].

Breast cancer continues to impose a substantial burden across Europe, with over 2.1 million new female cases in 2022 (age-standardized incidence~253 per 100,000) and marked geographic variation (incidence rates ranging from ~71 to 190 per 100,000 across EU countries), while age-adjusted mortality has declined by ~25% since 1990 yet remains a leading cause of cancer death in women—highlighting the timeliness of examining modifiable risk factors such as thyroid hormone therapy [11]. Given the increasing prescription rates of thyroid hormones and inconsistent findings from prior observational studies, a comprehensive synthesis of the evidence is both timely and warranted.

Although narrative reviews have discussed the potential relationship between thyroid hormone therapy and breast cancer, no contemporary systematic review and meta-analysis has comprehensively synthesized the available observational evidence using current evidence-based methodology. Therefore, the objective of this study is to systematically review and quantitatively synthesize observational studies examining the association between thyroid hormone treatment and breast cancer risk in women. By pooling data across diverse populations and study designs, and rigorously evaluating sources of heterogeneity and bias, this review aims to clarify the strength and consistency of the association and inform future research and clinical decision-making.

## 2. Materials and Methods

This systematic review and meta-analysis was conducted following PRISMA guidelines and registered in the PROSPERO database under the title “Is thyroid hormone therapy associated with a higher risk for the development of breast cancer? A systematic review and meta-analysis of observational studies” (PROSPERO ID: CRD42022348966).

### 2.1. Search Strategy and Information Sources

We developed a sensitive search strategy using medical subject headings (MeSH) and relevant keywords related to thyroid hormone treatment and breast cancer. The databases MEDLINE (via PubMed), EMBASE, and Web of Science were searched. Grey literature sources (including trial registries such as ClinicalTrials.gov, WHO ICTRP, and PROSPERO) were screened to identify potentially relevant studies but were not included in the quantitative synthesis. Additional records were identified through screening of reference lists from relevant articles, hand searches, and prospective registration platforms, including PROSPERO, ClinicalTrials.gov, WHO ICTRP, and the metaRegister of Controlled Trials.

The search terms included “thyroxine,” “thyroid hormones,” “liothyronine,” “levothyroxine,” and “breast cancer”.

**Figure 1:**
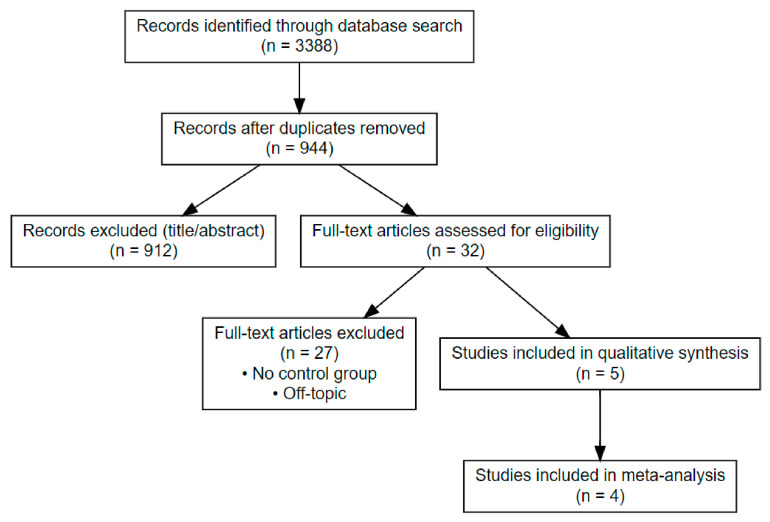
PRISMA Flow Diagram. A flow diagram showing the number of records identified, screened, excluded, and included in the final meta-analysis. Detailed reasons are provided in Supplementary Tables S2–S4.

Our systematic search in PubMed, EMBASE and Web of Science yielded 3388 studies. After removing duplicates, 944 records were screened by title and abstract. Of these, 917 were excluded (see criteria below). The full texts of the remaining 32 studies were assessed for eligibility. Of those, 27 were excluded for reasons including lack of a control group, irrelevance to the research question, or duplicate data. Two studies by Mustacci et al. used the same data set as Kapdi et al. but did not meet the inclusion criteria [12,13]. Ultimately, four case–control studies fulfilled all criteria and were included in the meta-analysis (Figure 1).

### 2.2. Selection Criteria

Original observational studies (cohort, case–control, or cross-sectional) published in English and involving adult female participants (aged ≥18 years) were eligible. Studies had to assess the association between thyroid hormone treatment and breast cancer incidence (Supplementary Tables S1–S3).

We excluded studies that were not original (e.g., reviews, meta-analyses, editorials, commentaries), studies not published in English, non-human studies, conference abstracts, and reports that focused on cancers other than breast cancer. Studies that did not report breast cancer incidence or failed to provide a comparison group were also excluded. If multiple reports from the same cohort were found, the most complete and recent report was retained (Supplementary Table S4). Titles and abstracts were screened independently by two reviewers (SK, JF), and disagreements were resolved by consensus or adjudication by a third reviewer (CP-K, MB).

### 2.3. Data Extraction and Quality Assessment

After deduplication using Mendeley 2.146.0, records were imported into Covidence systematic review software (Veritas Health Innovation, Melbourne, Australia; accessed/used in 2025) for screening and data extraction [14]. Full-text articles meeting eligibility criteria were assessed, and relevant data were extracted using a standardized Excel form. Extracted variables included study design, population size, participant demographics, thyroid treatment type, outcome measures, and effect estimates (odds ratios). Three reviewers independently extracted data and assessed study quality using the COSMOS-E guidance, focusing on population comparability, outcome definition, and risk of selection and information bias. Risk of bias was assessed using the COSMOS-E framework, which was specifically developed for observational etiological research and has been recommended for systematic reviews evaluating associations between exposures and health outcomes. Although COSMOS-E guidance was followed, a structured domain-based risk of bias assessment was limited by reporting inconsistencies across primary studies (Supplementary Tables S5–S7). For each eligible study, we extracted the adjusted effect estimate reported by the authors whenever available. Crude event counts are presented descriptively in Table 1 and Table 2 but were not used to calculate the pooled adjusted odds ratios.

Whenever available, we extracted the most fully adjusted effect estimate reported in the original publication. The OR-based meta-analysis was prespecified as the primary analysis because odds ratios were consistently reported across the eligible case–control studies. For the primary OR-based analysis, odds ratios were used to maintain consistency of the effect measure. For Wändell et al., the original publication reported adjusted HRs from Cox regression models, while breast cancer event counts by treatment status were obtained directly from the study authors [15]. Therefore, an unadjusted OR was calculated from these author-provided event counts for inclusion in the OR-based primary analysis. The adjusted HR from Wändell et al. was included exclusively in the qualitative synthesis [15].

Accordingly, no primary quantitative synthesis combining odds ratios and hazard ratios was performed. The adjusted HR from Wändell et al. was retained for qualitative comparison with the additional cohort study by Planck et al. [15,18]. Because only two studies reported adjusted HRs, no quantitative HR meta-analysis was performed.

### 2.4. Statistical Analysis

The primary meta-analysis was performed using a random-effects model with restricted maximum likelihood (REML) estimation. REML was selected because it provides an approximately unbiased estimate of the between-study variance (τ^2^), particularly when only a small number of studies are available. Confidence intervals for the pooled effect were calculated using the Hartung–Knapp adjustment, which has been recommended for random-effects meta-analyses with few studies because it provides more robust and conservative inference than the conventional DerSimonian–Laird approach. Given the substantial between-study heterogeneity and the limited number of included studies (k = 4), the random-effects model was prespecified as the primary analytical approach, whereas fixed-effect estimates were considered sensitivity analyses only. This approach was chosen a priori to account for anticipated clinical and methodological heterogeneity among the included observational studies [19,20]. Fixed-effects models were used only as sensitivity analyses [21].

For continuous outcomes (if applicable), weighted mean differences (WMDs) with 95% confidence intervals (CIs) were calculated. For dichotomous outcomes, odds ratios (ORs) with 95% CIs were pooled using inverse-variance weighting. A leave-one-out sensitivity analysis excluding the largest study (Wändell et al.) was prespecified to assess the robustness of the pooled estimates. Inconsistency and heterogeneity were assessed using the I^2^ statistic and τ^2^ estimate, respectively [15]. Values of I^2^ > 50% and >80% were interpreted as moderate and substantial heterogeneity, respectively [22]. Funnel plots and Egger’s regression test were used to assess publication bias. Statistical analyses were performed in collaboration with a biostatistician (A.H., Department of Biostatistics and Medical Biometry, Bielefeld University).

## 3. Results

A total of 221,254 patients were treated with thyroid hormones and compared with 4,385,666 untreated individuals. The sample sizes of the included studies ranged from 2414 (Shapiro et al.) to 4,271,551 (Wändell et al.) [15,16]. In total, 4686 breast cancer cases were identified in the treatment groups and 103,624 in the control groups. The quality of all four included studies was rated as good to very good according to the Guyatt et al. evaluation criteria [23].

Four studies fulfilled the eligibility criteria for the prespecified primary OR-based meta-analysis. One additional cohort study (Planck et al. [18]) met the eligibility criteria but reported adjusted hazard ratios (HRs) only and was therefore included exclusively as qualitative time-to-event evidence. Three studies (Kapdi, Shapiro, and Wu) reported odds ratios that were used directly in the primary quantitative synthesis [13,16,17]. In contrast, Wändell et al. reported only adjusted HRs in the original publication [15]. Breast cancer event counts stratified by levothyroxine treatment were obtained directly from the authors, allowing calculation of a crude OR for inclusion in the prespecified OR-based meta-analysis. The published adjusted HR from Wändell et al. was retained exclusively for qualitative comparison with the cohort study by Planck et al. [15,18]. Accordingly, odds ratios and hazard ratios were not quantitatively combined because these effect measures are not directly interchangeable.

Whenever available, the most fully adjusted odds ratio reported in the original publication was used for quantitative synthesis to minimize confounding bias. Accordingly, adjusted ORs were used for Kapdi et al., Shapiro et al., and Wu et al. [13,16,17]. Because no OR was reported by Wändell et al., a crude OR was calculated from author-provided event counts solely for the prespecified OR-based analysis, whereas the published adjusted HR was retained for qualitative comparison only [15]. The adjusted estimates extracted from the primary studies accounted for important covariates such as age, comorbidities, socioeconomic characteristics, healthcare utilization, hormone therapy use, reproductive factors, and cancer screening behaviour, depending on the original study design. A detailed summary of the adjustment variables is provided in Supplementary Table S6. Although raw event counts were available for all included studies, the use of adjusted estimates whenever possible reduces residual confounding and provides a more appropriate basis for quantitative synthesis of observational evidence.

**Figure 2:**
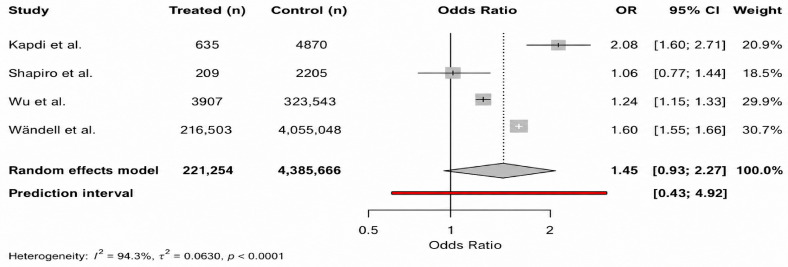
Forest plot of the association between thyroid hormone treatment and breast cancer risk using the prespecified random-effects model (REML with Hartung–Knapp adjustment). Fixed-effect estimates are provided in the Supplementary Material as sensitivity analyses only. Squares represent the effect estimate for each individual study, with square size proportional to study weight. Horizontal lines indicate 95% confidence intervals. The diamond represents the pooled random-effects estimate. The solid vertical line indicates the line of no effect (OR = 1), whereas the dotted vertical line indicates the pooled effect estimate. The red horizontal bar represents the 95% prediction interval [13,15,16,17].

We included four observational studies in the meta-analysis, comprising a total of 221,254 women receiving thyroid hormone treatment and 4,385,666 untreated controls (Table 1). The primary random-effects meta-analysis yielded a pooled OR of 1.45 (95% CI: 0.93–2.27). Although the point estimate suggested a possible epidemiological signal, the confidence interval crossed unity and the result was therefore not statistically significant, while between-study heterogeneity was substantial (I^2^ = 94.3%), indicating considerable variability across the included studies. Therefore, the pooled estimate should be interpreted with caution. Fixed-effect estimates are provided for completeness as a sensitivity analysis and are not considered the primary basis for inference because of the substantial between-study heterogeneity (Figure 2).

### 3.1. Funnel Plot and Publication Bias

**Figure 3:**
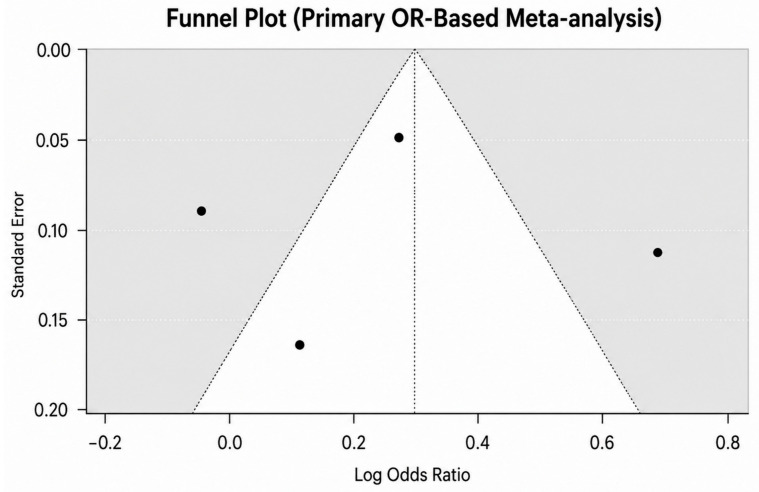
Funnel plot of the primary OR-based random-effects meta-analysis assessing potential publication bias. Each circle represents an individual study. The vertical dashed line indicates the pooled log odds ratio, whereas the diagonal dashed lines represent the pseudo 95% confidence limits expected in the absence of publication bias. The funnel plot should be interpreted with caution because only four studies were available for quantitative synthesis.

Visual inspection of the funnel plot did not reveal marked asymmetry; however, interpretation is inherently limited because only four studies were available. Egger’s regression test yielded an intercept of −2.09 (*p* = 0.054), but this result should not be interpreted as evidence for or against publication bias because statistical tests for funnel-plot asymmetry are underpowered when fewer than ten studies are available (Figure 3). Given the small number of included studies (k = 4), formal assessment of publication bias using funnel plots and Egger’s test is limited and should be interpreted with caution.

### 3.2. Leave-One-Out Sensitivity Analysis

To evaluate the robustness of our findings, we performed a leave-one-out sensitivity analysis. Exclusion of the largest study [15], which contributed the majority of participants, yielded a pooled OR of 1.40 (95% CI: 0.59–3.28) under the random-effects model. Although the confidence interval widened and crossed unity, the direction of the association remained positive. Similar findings were observed following sequential exclusion of each of the remaining studies (Table 3), indicating that no single study alone explained the overall direction of the pooled estimate, although substantial heterogeneity persisted.

The certainty and quality of the evidence was assessed using the GRADE framework (Supplementary Table S8). The certainty of evidence was rated as low because of serious inconsistency and serious imprecision due to high heterogeneity (I^2^ = 94.3%). Publication bias could not be reliably assessed because only four studies were available.

Between-study heterogeneity was substantial in the primary OR-based analysis (I^2^ = 94.3%; τ^2^ = 0.063). The 95% prediction interval was wide (OR 0.43–4.92), indicating that the true effect in a future comparable study could plausibly range from no association to a positive association. Leave-one-out analyses showed that the direction of the pooled estimate remained positive after sequential exclusion of each individual study, although confidence intervals remained wide and heterogeneity persisted. The pooled ORs ranged from 1.32 after exclusion of Shapiro et al. to 1.57 after exclusion of Kapdi et al. (Table 3) [13,16].

In addition to the four studies included in the primary meta-analysis, we identified a high-quality population-based cohort study by Planck et al. (2021) conducted in Sweden [18]. This study assessed the incidence of breast cancer in over 11,000 women treated with T3 and T4 thyroid hormones, using national registry data. The authors reported an adjusted hazard ratio (HR) of 0.94 (95% CI: 0.76–1.16), suggesting no statistically significant association between thyroid hormone therapy and breast cancer risk.

Because only two studies [15,18] reported adjusted hazard ratios derived from time-to-event analyses, whereas the remaining studies reported odds ratios from case–control designs, a pooled HR meta-analysis was not considered methodologically appropriate. Therefore, quantitative synthesis was restricted to the prespecified OR-based analysis.

Nevertheless, both nationwide Swedish cohort studies independently evaluated the association using Cox proportional hazards models [15]. reported a modest but statistically significant association between levothyroxine treatment and incident breast cancer (adjusted HR 1.09, 95% CI 1.04–1.14), whereas Planck et al. observed no statistically significant increase in breast cancer risk among thyroid hormone users (adjusted HR 0.94, 95% CI 0.76–1.16) [18]. Although these studies differed in exposure definition and adjustment strategy, they provide important complementary time-to-event evidence that should be interpreted qualitatively rather than quantitatively.

## 4. Discussion

This systematic review and meta-analysis aimed to investigate the association between thyroid hormone therapy and the risk of developing breast cancer in women. Overall, the available observational evidence suggests a possible epidemiological signal between thyroid hormone treatment and breast cancer risk, with a pooled odds ratio (OR) of 1.45 (95% CI: 0.93–2.27) using a random-effects model, but not reaching statistical significance. The random-effects model was considered the primary analytical approach because of the substantial between-study heterogeneity. Fixed-effect analyses were performed solely as sensitivity analyses and are therefore not interpreted further. Given the observational nature of the included studies, the present findings should be regarded as hypothesis-generating rather than confirmatory and should not be interpreted as evidence of a causal relationship. While the absolute risk increase for individual patients may be modest, the high prevalence of thyroid hormone use in the general population amplifies the potential public health relevance of this association [15,17]. These findings suggest a possible epidemiological signal between thyroid hormone therapy and breast cancer risk, although residual confounding cannot be excluded.

The biological plausibility of this association is supported by mechanistic studies showing that thyroid hormones can influence cellular proliferation, apoptosis, and oestrogen-like effects through thyroid hormone receptors, activation of MAPK and PI3K and other signalling pathways [16,18]. In breast tissue, they can interact with oestrogen receptor α (ERα), enhancing estrogenic responses even in the absence of oestrogen. These effects may be mediated via thyroid hormone receptor α and non-genomic mechanisms [24]. Levothyroxine (L-T4), the most commonly prescribed form of thyroid hormone replacement, may enhance tumour growth under certain conditions by mimicking or amplifying estrogenic activity in breast tissue [13,25]. Although several experimental studies provide biological plausibility for an association between thyroid hormone signalling and breast carcinogenesis, these mechanistic observations should not be interpreted as evidence of causality in humans. The epidemiological findings remain observational and cannot distinguish the effects of thyroid hormone therapy from those of the underlying thyroid disease or residual confounding.

Our analysis included four large observational studies with over 4 million controls and more than 220,000 patients treated with thyroid hormones. While the observational nature of these studies precludes causal inference, the strength of our meta-analysis lies in the size and consistency of the effect direction. However, the inconsistency was high (I^2^ = 94.3%), suggesting significant variability between studies. The high heterogeneity observed reflects substantial differences in study design, populations, confounder adjustment, or thyroid hormone dosage and duration and reduces confidence in the pooled estimate. With only four OR-based studies available, exploration of potential sources of heterogeneity—such as exposure definition, menopausal status, screening intensity, or duration and dose of therapy—was not feasible. Accordingly, these findings should be interpreted as hypothesis-generating rather than causal, which was the primary reason for downgrading certainty of evidence in the GRADE assessment.

The pooled estimate was heavily influenced by a single large national registry-based study [17], which contributed the majority of participants. Although sensitivity analyses excluding this study were performed, the limited number of remaining studies restricts the robustness of alternative pooled estimates and underscores the reliance on registry-level data. Sensitivity analysis excluding the largest study yielded a pooled OR of 1.40 (95% CI: 0.59–3.28) with I^2^ = 87.8%, indicating persistence of the direction of effect, although substantial heterogeneity remained [15]. No firm conclusions regarding publication bias can be drawn because only four studies contributed to the primary meta-analysis, making both funnel plots and Egger’s regression test inherently unreliable. According to the GRADE assessment, the overall certainty of evidence was rated as low, downgraded primarily due to serious heterogeneity, imprecision and inconsistency [23,26,27].

An additional consideration is that the two nationwide Swedish cohort studies reported adjusted hazard ratios rather than odds ratios. Because hazard ratios derived from Cox proportional hazards models are not directly interchangeable with odds ratios obtained from case–control studies, these results were not quantitatively pooled. Nevertheless, the cohort studies provide valuable complementary evidence based on time-to-event analyses [15]. reported a modest but statistically significant association, whereas Planck et al. found no significant increase in breast cancer risk [18]. represents one of the largest contemporary registry evaluating T3/T4 replacement and therefore constitutes an important complementary source of evidence despite not being quantitatively pooled. These differences may reflect variations in study populations, exposure definitions, adjustment strategies, and duration of follow-up, highlighting the need for further large prospective cohort studies using harmonized methodologies.

The study highlights that the indication for thyroid hormone treatment must be carefully assessed. The increasing prescription of thyroid hormone preparations without a comparable increase in the incidence of thyroid disease suggests that there is significant overtreatment [28,29]. This leads to considerable additional costs in the healthcare system. The increase in prescriptions in the US between 1997 and 2016 was accompanied by a corresponding tripling of costs for the healthcare system (increase from $1.1 billion to $3.2 billion). It is striking that the increase in prescriptions for thyroid hormones particularly affects the older age group [29]. It has been shown that, especially in this age group, even slightly elevated TSH levels do not benefit health through therapy with L-thyroxine [30]. With an increasing incidence of breast cancer, which may be associated with (unnecessary) thyroid hormone intake, significant additional costs for the diagnosis and treatment of breast cancer patients are to be expected. These observations reinforce existing guideline recommendations that thyroid hormone replacement should be prescribed only when clinically indicated rather than suggesting any change in current clinical practice based on the present findings [31,32].

The five included studies were conducted across diverse geographic settings—North America, Europe, Asia, and Africa/Latin America—which supports the generalizability of the observed association between thyroid hormone therapy and breast cancer risk across health systems and ethnic populations.

Some limitations must be acknowledged. First, all included studies were observational, and residual confounding cannot be ruled out. Second, detailed exposure assessment methods varied across studies, and misclassification may have occurred. Important variables such as type of thyroid hormone (e.g., levothyroxine vs. combination therapy), dose, duration of treatment, cumulative exposure, and recency of use were inconsistently reported or unavailable. This precludes dose–response analyses and limits clinical interpretability of the findings.

Similarly, information on circulating thyroid hormone concentrations, TSH levels, treatment adherence, cumulative dose, duration of therapy, menopausal status, and tumour receptor subtype was inconsistently reported across the included studies, precluding meaningful subgroup analyses. Third, differences in populations (e.g., age, menopausal status, iodine sufficiency) may influence generalizability. It should also be noted that the observed proportion of breast cancer cases among treated individuals (2.24%) reflects study-specific follow-up periods rather than lifetime risk, and therefore should not be directly compared with population-level lifetime incidence estimates (12–13%). Stratified analyses by age (e.g., <50 vs. ≥50 years) were not feasible due to insufficient reporting in the primary studies, although age and menopausal status are likely important effect modifiers and should be addressed in future research. Fourth, we did not stratify by duration of therapy or TSH levels, which may modulate cancer risk. Fifth, there is no differentiation between oestrogen-receptor positive or negative tumours (e.g., ER-positive vs. ER-negative) as hormone sensitivity could contribute to difference in the frequency between these tumour types as described above, offering critical insight into hormone-related carcinogenesis.

Finally, we could not stratify results by subclinical versus overt hypothyroidism due to insufficient reporting in the primary studies. Residual confounding and detection bias remain central concerns. Women receiving thyroid hormone treatment may differ systematically from untreated individuals in healthcare utilization, comorbidity burden, and cancer screening practices. Such biases cannot be adequately addressed using aggregate-level meta-analysis of observational studies and may partially explain the observed association.

Nonetheless, this meta-analysis provides a comprehensive synthesis of current evidence. Our results indicate a potential risk signal that should prompt further investigation. Importantly, thyroid hormone replacement therapy is frequently prescribed for subclinical hypothyroidism or nonspecific symptoms, raising concerns about overtreatment [33,34,35]. Future studies should differentiate between treatment of overt and subclinical thyroid dysfunction in relation to breast cancer risk and prioritize prospective cohort designs with standardized hormone exposure assessment, control for oestrogen exposure and menopausal status, and explore dose–response relationships as well as hormone receptor status of breast tumours.

Previous narrative reviews have summarized the biological plausibility linking thyroid hormone signalling with breast carcinogenesis and discussed the potential clinical implications of thyroid hormone therapy [36,37]. However, these reviews did not systematically synthesize the available epidemiological evidence or quantitatively estimate the association between thyroid hormone treatment and breast cancer risk. Therefore, the present study addresses this important gap by providing a systematic review and meta-analysis of observational studies.

Given the observational nature of the evidence, these findings should not alter current indications for thyroid hormone replacement. Rather, they highlight the need to avoid unnecessary overtreatment and to further investigate whether specific patient subgroups may differ in breast cancer risk.

## 5. Conclusions

Our meta-analysis of 221,254 women suggests that therapy with thyroid hormones is associated with a potential epidemiological signal linking thyroid hormone treatment and breast cancer risk (OR = 1.45, 95% CI: 0.93–2.27). However, because the pooled estimate was not statistically significant, heterogeneity was substantial, and residual confounding cannot be excluded, these findings should be regarded as hypothesis-generating rather than confirmatory. Future large prospective studies with standardized exposure assessment and comprehensive adjustment for relevant confounders are needed before causal or clinical conclusions can be drawn.

## Figures and Tables

**Table 1:** Characteristics of studies included in the primary random-effects meta-analysis and extracted effect estimates. This table summarizes the number of patients treated with thyroid hormones and those not treated, along with the corresponding number of breast cancer cases reported in each group. The data were extracted from four observational case–control studies included in the primary meta-analysis. Event counts for the Wändell et al. study were obtained through author correspondence [15].

**Study**	**Design**	**Country**	**Treated (*n*)**	**Controls (*n*)**	**Events (Treated/Control)**	**Effect Estimate Used**	**Adjusted Estimate Available**	**Entered Analysis**
[13]	Case–control	USA	635	4870	77/303	Adjusted OR	Yes	Primary OR
[16]	Case–control	USA	209	2205	60/635	Adjusted OR	Yes	Primary OR
[17]	Case–control	Taiwan	3907	323,543	916/64,570	Adjusted OR	Yes	Primary OR
[15]	Cohort	Sweden	216,503	4,055,048	3633/42,802	Crude OR calculated from author-provided counts	Not for primary OR analysis	Primary OR/Secondary HR

Footnote: Event counts are presented descriptively. Quantitative synthesis was performed using the adjusted effect estimates reported by each study. Details of extracted effect estimates and adjustment variables are provided in Supplementary Table S1. Breast cancer event counts are presented for descriptive purposes only and should not be interpreted as the basis for the pooled effect estimates. For studies reporting multiple effect estimates, the estimate used for quantitative synthesis is indicated. Where no OR was reported (Wändell et al.), a crude OR was calculated from author-provided event counts for the primary OR-based meta-analysis, whereas the published adjusted HR was retained for qualitative comparison only [15].

**Table 2:** Effect estimates extracted from the included studies and their use in the quantitative and qualitative evidence synthesis.

**Study**	**Effect Measure Extracted**	**Estimate (95% CI)**	**Effect Estimate Used for Meta-Analysis**	**Covariates**	**Analysis**
[13]	Crude OR (calculated from published counts)	2.08 (1.60–2.71)	Adjusted OR	Age	Primary OR
[16]	Age-standardized RR (published) */Crude OR (calculated) **	1.06 (0.77–1.44)	Age-standardized	Age	Primary OR
[17]	Adjusted OR	1.24 (1.15–1.33)	Fully adjusted logistic regression	Age, comorbidities, healthcare utilization, medications	Primary OR
[15]	Crude OR (primary OR meta-analysis) Adjusted HR (qualitative evidence only) *	1.60 (1.55–1.66) 1.09 (1.04–1.14) *	Crude (calculated by authors) Fully adjusted Cox model	Age, education, immigrant status, marital status, neighborhood deprivation, obesity, diabetes, COPD, alcoholism, liver disease, biliary/pancreatic disease, inflammatory polyarthropathies, kidney disease, inflammatory diseases of female pelvic organs	Primary OR Secondary HR
[18]	Adjusted HR (qualitative evidence only) *	0.94 (0.76–1.16) *	Fully adjusted Cox model	Age, sex, previous thyroid cancer, previous cancer, antithyroid drug use, sex hormone use, dose	Secondary HR

* Published adjusted hazard ratio (HR) extracted from the original study. HRs were retained for qualitative comparison only because hazard ratios and odds ratios were not quantitatively pooled. ** Crude odds ratio (OR) calculated by the authors from published raw event counts because no OR was reported in the original publication.

**Table 3:** Leave-one-out sensitivity analysis of the primary OR-based random-effects meta-analysis. Each study was omitted sequentially to evaluate its influence on the pooled odds ratio and between-study heterogeneity. The table reports the estimated between-study variance (τ^2^), pooled odds ratio (OR), and corresponding 95% confidence interval (CI) after omission of each individual study. The pooled effect remained directionally consistent across all analyses, indicating that no single study alone explained the overall findings. Confidence intervals widened after exclusion of individual studies, reflecting the limited number of available studies and persistent between-study heterogeneity.

**Excluded Study**	**τ^2^**	**Pooled OR**	**95% CI**
Excluding Kapdi	0.054	1.57	0.91–2.71
Excluding Shapiro	0.034	1.32	0.85–2.06
Excluding Wu	0.085	1.54	0.78–3.02
Excluding Wändell	0.101	1.40	0.59–3.28

## Data Availability

All data used in this systematic review and meta-analysis are derived from published studies and publicly available sources cited in the manuscript. No new individual participant data were generated for this study. Extracted data and analytical code used to generate the results are available from the corresponding author upon reasonable request.

## Supplementary Materials

The following supporting information can be downloaded at: https://www.mdpi.com/article/10.3390/metabo16070465/s1, Supplementary Table S1. Characteristics of included studies and effect estimates extracted for quantitative synthesis, Supplementary Table S2. Characteristics of the included studies after full-text acquisition, Supplementary Table S3. Characteristics of the excluded studies after full-text acquisition, Supplementary Table S4. Reasons for exclusion for full-text articles assessed for eligibility, Supplementary Table S5. GRADE assessment of the certainty of evidence, Supplementary Table S6. Adjusted Covariates/Confounders in Included Studies. Most included studies contributed adjusted effect estimates. For Wändell et al., a crude OR was calculated from author-provided event counts for the primary OR-based analysis, whereas the published adjusted HR is presented qualitatively [15], Supplementary Table S7. Risk of bias assessment according to the COSMOS-E guidance, Supplementary Table S8. GRADE Summary of Evidence Table. GRADE assessment tables summarizing judgments across five domains of evidence quality.
