# Supplementary material for: Thyroid Hormone Treatment and Breast Cancer Risk in Women: A Systematic Review and Meta-Analysis of Observational Studies
Source: Metabolites. 2026 Jul 2;16(7):465. doi: 10.3390/metabo16070465 (PMC13413771; doi:10.3390/metabo16070465)
Supplement: Supplementary file 1 [file metabolites-16-00465-s001.zip › metabolites-4391656-supplementary.pdf]

**Supplementary material**

In MEDLINE, the following Boolean search string was applied in MEDLINE on 15 February 2025:

((((((((Thyroxine[Title/Abstract]) ) OR (Thyroid hormones[Title/Abstract])) OR (Liothyronine[Title/Abstract])) OR (L-Thyroxine[Title/Abstract])) OR (thyroid supplements[Title/Abstract])) OR (tri-iodthyronine[Title/Abstract])) OR (levothyroxine[Title/Abstract])) AND ((breast cancer[Title/Abstract]) OR (breast neoplasms[MeSH Terms]))

**Table S1.** Characteristics of included studies and effect estimates extracted for quantitative synthesis.

| Study | Country | Study design | Data source | Study population | Exposure definition | Comparator | Follow-up | Breast cancer outcome | Effect estimate extracted | Covariate adjustment | Included |
|---|---|---|---|---|---|---|---|---|---|---|---|
| Kapdi et al., 1976 | USA | Case-control | Hospital-based | Women with breast cancer and controls | Thyroid hormone treatment | No thyroid hormone treatment | NR | Incident breast cancer | Adjusted OR 2.08 (95% CI 1.60–2.71) | Age (if applicable; according to original publication) | Primary OR meta-analysis (adjusted model) |
| Shapiro et al., 1980 | USA | Case-control | Population-based | Women with breast cancer and controls | Thyroid hormone treatment | No thyroid hormone treatment | NR | Incident breast cancer | Adjusted OR 1.06 (95% CI 0.77–1.44) | Age | Primary OR meta-analysis (adjusted model) |
| Wu et al., 2018 | Taiwan | National population-based case-control | Taiwan National Health Insurance Research Database | Women with newly diagnosed breast cancer | Levothyroxine use before index date | No levothyroxine use | Registry-based | Incident breast cancer | Adjusted OR 1.24 (95% CI 1.15–1.33) | Age, comorbidities, healthcare utilization, medications (original multivariable model) | Primary OR meta-analysis (fully adjusted multivariable model) |
| Wändell et al., 2020 | Sweden | National population-based cohort | Swedish National Registers | Adults ≥18 years without previous cancer | ≥2 dispensed prescriptions of levothyroxine during 2005–2006 | No levothyroxine treatment | Mean ≈6 years (2009–2015) | Incident breast cancer | Crude OR (calculated from author-provided counts) and adjusted HR 1.09 (95% CI 1.04–1.14) | Age, education, immigrant status, marital status, neighborhood deprivation, obesity, diabetes, COPD, alcoholism, liver disease, biliary/pancreatic disease, inflammatory polyarthropathies, kidney disease, inflammatory diseases of female pelvic organs | Primary OR/ Secondary qualitative evidence |
| Planck et al., 2020 | Sweden | National registry-based cohort | Swedish Prescribed Drug Register + Cancer Register + Cause of Death Register | Adults receiving thyroid hormone replacement | ≥3 purchases of liothyronine (alone or combined with LT4) | LT4-only users | Median 8.1 years | Incident breast cancer | Adjusted HR 0.94 (95% CI 0.76–1.16) | Age, sex (where applicable), previous thyroid cancer, previous cancer, antithyroid drug use, sex hormone use, dose | Secondary qualitative evidence (fully adjusted HR model) |

Abbreviations: OR, odds ratio; HR, hazard ratio; COPD, chronic obstructive pulmonary disease; LT4, levothyroxine. Footnote: Breast cancer event counts presented in Table 1 are descriptive only and were not used for quantitative synthesis. Whenever available, the most fully adjusted effect estimate reported by the original study was extracted. Odds ratios and hazard ratios were analyzed separately because these measures are not directly interchangeable.

**Table S2.** Characteristics of the included studies after full-text acquisition.

| First Author, Year, Country | Study Design, Source of Data | Study Population | Participants' Characteristics |
|---|---|---|---|
| Kapdi, 1976, USA | Retrospective cohort; Mammography clinic records | Women attending breast cancer screening (N=5,555) | Women aged predominantly 40+, with and without thyroid supplementation |
| Shapiro, 1980, Multi-country (Africa, Latin America, Israel) | Multicenter case-control; Hospital interviews and patient histories | Women <70 years old with newly diagnosed breast cancer | Cases and controls interviewed for drug use history including thyroid hormone; matched for age, hospital, region |
| Wu, 2021, Taiwan | Population-based case-control; Taiwan National Health Insurance Research Database (NHIRD) | Women ≥20 years old with newly diagnosed breast cancer (2001–2011) | Levothyroxine users and matched non-users; matched by age, income, urbanization |
| Wändell, 2020, Sweden | Retrospective cohort; Swedish National Registers (Prescribed Drug, Cancer, and Patient Register) | Adults ≥18 years with ≥2 prescriptions of levothyroxine and no prior cancer | Long-term levothyroxine users; adjusted for comorbidities, socioeconomic and region data |
| Planck, 2020, Sweden | Retrospective cohort; Swedish Prescribed Drug Register and Cancer Register | Women with levothyroxine prescriptions between 2006–2012 | Stratified by age and treatment duration; no cancer at baseline; breast cancer incidence tracked through registry |

**Table S3.** Characteristics of the excluded studies after full-text acquisition.

| First Author | Year | Title | Study Design | Reason for Exclusion |
|---|---|---|---|---|
| Chan | 2017 | Lower TSH and higher free thyroxine predict incidence of prostate but not breast, colorectal or lung cancer | Prospective cohort | No thyroid hormone treatment exposure; focused only on hormone levels. Breast cancer not separately analyzed for therapy risk. |
| Danielson | 1982 | Non- estrogenic drugs and breast cancer | Nested case-control | Included multiple non-estrogenic drugs; could not isolate thyroid hormone treatment. No therapy-specific ORs. |
| Kuijpens | 2005 | Hypothyroidism might be related to breast cancer in post-menopausal women | Nested case-control | Assessed endogenous thyroid hormones without treatment data; no risk estimates for therapy. |
| Leese | 2016 | Liothyronine use in a 17 year observational population-based study - the TEARS study | Retrospective cohort | Did not isolate levothyroxine users or stratify cancer incidence by treatment; breast cancer not independently assessed. |
| Leese | 1992 | Morbidity in patients on L-thyroxine: a comparison of those with a normal TSH to those with a suppressed TSH | Registry-based retrospective study | Focused on cardiovascular and bone morbidity; breast cancer data not provided. |
| Lei | 2022 | Free triiodothyronine and free thyroxine hormone levels in relation to breast cancer risk: a meta-analysis | Meta-analysis | Meta-analysis of FT3/FT4 levels in breast cancer cases; not based on thyroid hormone therapy exposure. |
| Mustacchi | 1977 | Thyroid supplementation for hypothyroidism. An iatrogenic cause of breast cancer? | Ecological analysis | Ecological data; no control group or individual follow-up; insufficient for causality or pooled analysis. |
| Nisman | 2021 | Elevated Free Triiodothyronine Is Associated with Increased Proliferative Activity in Triple-negative Breast Cancer | Cross-sectional | Studied FT3 and tumor biology (TK1) in breast cancer patients; no incidence or treatment data. |
| Ortega-Olvera | 2018 | Thyroid hormones and breast cancer association according to menopausal status and body mass index | Case-control | Focused on hormone levels in BC stratified by BMI and menopausal status; did not assess therapy. |
| Rose | 1978 | Plasma thyroid-stimulating hormone and thyroxine concentrations in breast cancer | Cross-sectional | Cross-sectional T3/T4 measurement studies; no treatment data or prospective outcome modeling. |
| Saraiva | 2005 | Profile of thyroid hormones in breast cancer patients | Cross-sectional | Profiled thyroid hormones in existing BC patients; lacked treatment or risk data. |
| Shi | 2014 | Relationship between breast cancer and levels of serum thyroid hormones and antibodies: a meta-analysis | Meta-analysis | Meta-analysis of hormone/antibody levels; no treatment exposure or risk estimates. |
| Sibio | 2014 | Triiodothyronine and breast cancer | Observational descriptive | Lacked control group and risk modeling; descriptive T3 levels only. |

| | | | | |
|---|---|---|---|---|
| Tosovic | 2012 | Prospectively measured thyroid hormones and thyroid peroxidase antibodies in relation to breast cancer risk | Prospective cohort | Studied antibodies and hormone levels; no treatment or risk-based subgrouping. |
| Tran | 2022 | The effect of thyroid dysfunction on breast cancer risk: an updated meta-analysis | Meta-analysis | Meta-analysis, not original data; not usable as an independent study. |
| Tureken | 2014 | Thyroid disorders and breast cancer: a case report | Case report | Case report; single patient observation. Not suitable for meta-analysis. |
| Voutsadakis | 2022 | The TSH/Thyroid Hormones Axis and Breast Cancer | Narrative review | Narrative review; no original data or risk estimates. |
| Weng | 2019 | Breast Cancer Risk in Postmenopausal Women with Medical History of Thyroid Disorder | Cohort study | Reported pooled cancer risk; breast cancer estimates not separately extractable. |
| Yang | 2020 | Hyperthyroidism is associated with breast cancer risk and mammographic and genetic risk predictors | Prospective cohort | Focused on mammographic density and genetic risk, not thyroid therapy. |
| Yuan | 2020 | Causal associations of thyroid function and dysfunction with overall, breast and thyroid cancer | Mendelian randomization | Mendelian randomization study; used genetic proxies, not treatment data. |
| Zumoff | 1981 | Plasma levels of thyroxine and triiodothyronine in women with breast cancer | Cross-sectional | Biochemical hormone profile in cancer patients; no exposure-outcome structure. |
| Huang | 2021 | Risk of Breast Cancer in Females With Hypothyroidism | Population-based cohort | Methods and outcome definitions could not be verified. |
| Michalaki | 2009 | Breast cancer in association with thyroid disorders | Retrospective cross-sectional | Unclear treatment stratification and study design. |
| Rasmusson | 1987 | Thyroid function in patients with breast cancer | Cross-sectional | Descriptive hormone function study in BC patients; lacked control or risk estimates. |
| Strain | 1997 | Thyroid hormones and selenium status in breast cancer | Cross-sectional | Cross-sectional assessment of hormone and selenium status; no incidence or therapy data. |
| Takatani | 1989 | Relationship between the levels of serum thyroid hormones or estrogen status and the risk of breast cancer genesis in Japanese women | Case-control | Inconsistent treatment data and no risk modeling were present. |
| Vorherr | 1978 | Thyroid disease in relation to breast cancer | Narrative review | Narrative review without original patient data or effect estimates. |

**Table S4.** Reasons for exclusion for full-text articles assessed for eligibility.

| Reason for exclusion | Number of studies (n) |
|---|---|
| No thyroid hormone treatment exposure / endogenous hormone levels only | 9 |
| Review, meta-analysis, commentary, or case report | 7 |
| No breast cancer incidence/risk estimate or outcome not extractable | 5 |
| Thyroid disorder exposure without separable treatment effect | 3 |
| Duplicate or overlapping dataset | 1 |
| Genetic proxy / Mendelian randomization | 1 |
| Cross-sectional biomarker/tumor biology only | 1 |

**Table S5**. GRADE assessment of the certainty of evidence.

**GRADE assessment of the certainty of evidence**

| Domain | Judgment | Downgrade? |
|---|---|---|
| **Risk of Bias** | No serious limitations overall; residual confounding cannot be excluded. | No |
| **Inconsistency** | Serious heterogeneity ($I^2$ = 94.3%). | Yes |
| **Indirectness** | Direct evidence for the research question. | No |
| **Imprecision** | Serious imprecision; 95% CI crosses the null (OR 1.43, 95% CI 0.90–2.28). | Yes |
| **Publication Bias** | Cannot be reliably assessed because only four studies were included. | No* |

**Overall certainty of evidence: LOW**

Downgraded for inconsistency and imprecision.

** Publication bias was not downgraded because it could not be reliably assessed with only four studies rather than because bias was absent.*

**Table S6.** Adjusted Covariates/Confounders in Included Studies**.** Most included studies contributed adjusted effect estimates. For Wändell et al., a crude OR was calculated from author-provided event counts for the primary OR-based analysis, whereas the published adjusted HR is presented qualitatively [15].

| Study | Year | Study Type | Adjusted Covariates / Confounders |
|---|---|---|---|
| Kapdi et al. | 1976 | Case-Control | Age |
| Shapiro et al. | 1980 | Case-Control | Age, race, education, marital status, reproductive history (e.g., parity, age at first birth) |
| Wu et al. | 2018 | Case-Control | Age, comorbidities (Charlson Comorbidity Index), income level, urbanization level, hormone replacement therapy use |
| Wändell et al. | 2020 | Cohort | Age, socioeconomic status, comorbidities, cancer screening history (proxy for surveillance bias), medication use |
| Planck et al. | 2021 | Cohort | Age, comorbidities, calendar year, level of care utilization, type and duration of thyroid hormone use |

**Table S7.** Risk of bias assessment according to the COSMOS-E guidance.

| Study | Selection Bias | Exposure Assessment | Outcome Assessment | Confounding | Selective Reporting | Overall Risk of Bias |
|---|---|---|---|---|---|---|
| Kapdi et al., 1976 | Moderate – Hospital-based case-control design may limit representativeness of the source population. | Moderate – Thyroid hormone exposure was obtained from medical records; dose and treatment duration were unavailable. | Low – Breast cancer diagnosis was clinically confirmed. | High – Limited adjustment for potential confounders; important variables (BMI, menopausal status, HRT, screening behaviour) were unavailable. | Low – Outcomes and analyses were reported as pre-specified. | Moderate |
| Shapiro et al., 1980 | Moderate – Population-based case-control study with appropriate control selection but limited participation data. | Moderate – Exposure obtained from medical records/interviews; treatment duration not consistently available. | Low – Histologically confirmed breast cancer. | High – Adjustment was limited primarily to age; residual confounding likely. | Low | Moderate |

| | | | | | | |
|---|---|---|---|---|---|---|
| Wu et al., 2018 | Low – Nationwide population-based database with predefined inclusion criteria. | Low – Levothyroxine prescriptions identified through a comprehensive national prescription registry. | Low – Incident breast cancer identified using validated national registry data. | Moderate – Multivariable adjustment performed; however, residual confounding (BMI, reproductive factors, family history, screening behaviour) cannot be excluded. | Low | Low |
| Wändell et al., 2020 | Low – Nationwide Swedish primary care cohort with large representative population. | Low – Thyroid hormone treatment identified from electronic health records and prescription data. | Low – Breast cancer diagnoses obtained from national health registers. | Moderate – Extensive adjustment performed, but residual confounding by indication and disease severity remains possible. | Low | Low |

**Table S8.** GRADE Summary of Evidence Table. GRADE assessment tables summarizing judgments across five domains of evidence quality.

| **Outcome** | **No. of Studies** | **Study Design** | **Risk of Bias** | **Inconsistency** | **Indirectness** | **Imprecision** | **Publication Bias** | **Effect Estimate (95% CI)** | **Certainty** | **Comments** |
|---|---|---|---|---|---|---|---|---|---|---|
| Breast cancer incidence (OR-based) | 4 | Case-control (Obs.) | Not serious | Serious | Not serious | Serious | Cannot be reliably assessed | OR 1.43 (95% CI: 0.90–2.28); $I^2 = 94.3\%$ | ⊕⊕◯◯ Low | Downgraded for serious inconsistency and serious imprecision. Publication bias could not be reliably assessed because only four studies were available. |
